# Influencing mechanism of the use behavior of clinical practice guidelines on antimicrobials: evidence from the integration of theory of reasoned action and organizational readiness for change

**DOI:** 10.1186/s12911-022-02019-w

**Published:** 2022-10-26

**Authors:** Junbin Huang, Wenbin Liu, Yimin Huang

**Affiliations:** Department of Health Management, School of Public Health, Fujian Medical University, Room 108 in the Building for School of Public Health, 1 Xuefubei Road, Minhou District, Fuzhou, 350122 Fujian China

**Keywords:** Clinical practice guidelines, Antimicrobials, Organizational readiness for change, Theory of reasoned action, Structural equation modeling

## Abstract

**Background:**

To confront the serious challenge of antimicrobial resistance, using clinical practice guidelines (CPGs) standardizing the prescription behavior is vital. However, the overall mechanisms remains largely unknown as to how guidelines' use behavior can be improved. This study aimed to identify the determinants and investigate their relationship to bridge the knowledge gap of overall influencing mechanism of the use behavior of CPGs on antimicrobials.

**Methods:**

By integrating theory of reasoned action (TRA) and organizational readiness for change (ORC), a structured questionnaire was developed to cover potential determinants that affect physicians’ use behaviors of CPGs on antimicrobials at the individual-level (attitude, subjective norm, and behavioral intention) and organizational-level (top management support and organizational resource allocation). A multi-stage random sampling was implemented to collect data from physicians in secondary and tertiary hospitals from eastern, central and western China. Structural equation model (SEM) was used to test the proposed hypotheses, and to analyze the relationship and mechanism among the factors.

**Result:**

In total, 815 physicians were included. Most physicians demonstrated a positive tendency toward the use of CPGs on antimicrobials, with a mean score of 3.95 (SD = 0.70). The reliability and validity analysis showed the questionnaire constructed from the integrated theoretical model of TRA and ORC was acceptable. The SEM validation results also showed that the top management support (*β* = 0.688, *P* < 0.001), organizational resource allocation (*β* = 0.129, *P* < 0.001), individual attitudes (*β* = 0.164, *P* < 0.001), subjective norms (*β* = 0.322, *P* < 0.001), and behavioral intentions (*β* = 0.424, *P* < 0.001) were positively associated with physicians’ use behaviors of CPGs on antimicrobials. Besides, top management support, organizational resource allocation, attitudes and subjective norms showed their mediating effects on regarding use behavior, which was 0.305, 0.129, 0.164 and 0.201, respectively.

**Conclusions:**

This study revealed the influence mechanism of the use of CPGs on antimicrobials from the individual and organizational perspectives. These findings will not only help formulate future strategies to promote the use of CPGs on antimicrobials, but also provide clues for more effective prescription interventions.

**Supplementary Information:**

The online version contains supplementary material available at 10.1186/s12911-022-02019-w.

## Background

As one of the most outstanding developments in the field of medical care, antimicrobials have been playing an important role in dramatically reducing the morbidity and mortality of bacterial infections [1, 2]. However, with its wider use, the emergence of antimicrobial resistance (AMR) has become common, which results in increased burden of bacterial infectious diseases and further caused multiple negative consequences [3–5]. In 2016, more than 700,000 deaths worldwide were associated with AMR, and without intervention, it was predicted that the number will exceed 10 million annually in 2050, which would greatly impair the sustainable development of the economy and society [6, 7].

To address the serious problems caused by inappropriate use of antimicrobials, many countries around the world have established antimicrobials stewardship (AMS) and taken multiple measures for containment of AMR, including guidelines, regulations, monitoring networks, curriculum training and so on. Among these measures, Clinical Practice Guidelines (CPGs) on antimicrobials have received widespread attention and paid much importance to. Since most of the CPGs are created basing on current best evidence from systematic reviews, randomized controlled trials and so on [8–10], they are intended to produce optimal health outcome, reduce inappropriate clinical care variation and minimize harm for the patients. Especially for CPGs on antimicrobials, previous studies held in many countries have demonstrated that the increasing compliance of CPGs on antimicrobials seems to reduce inappropriate prescription of antimicrobials, which would benefit the containment of AMR [11, 12]. And in the Global strategy for containment of antimicrobial resistance issued by WHO in 2001, development and dispense of CPGs on antimicrobials, compliance and use of CPGs on antimicrobials were listed in the action requirements for medical institutions and physicians, respectively [13]. Furthermore, it is also needed at the national level to establish and regularly update standardized treatment guidelines.

However, the CPGs on antimicrobials are not immediately effective in reducing inappropriate prescription of antimicrobials and regulating medical practices. For example, in China, one of the countries with the largest antimicrobials consumption especially in the tertiary and secondary hospitals of its health system, the Guiding Principles for Clinical Application of Antimicrobials have been issued since 2015 with the AMS main interventions of audit and monitoring, education and training of physicians' prescribing behavior, even taking advantage of the national policy context with various financial and economic incentives to reduce inappropriate prescribing, such as implementation of the zero-margin drug policy in tertiary and secondary hospitals, reform in medical insurance payment system, and so on [9, 14–18]. However, the per capita use of antibacterials was still much higher than the international level of 30% [16], and the low adherence of CPGs on antimicrobials was often reported in previous studies [19, 20]. Multiple sets of factors were considered as the causes of this phenomenon, such as misunderstanding of regarding CPGs’ importance, low intention to use CPGs in clinical practice, and so on at the physician level; lack of providing medication guidance or related training, inadequate prescription monitor and feedback, and so on at the hospital level [21]. Also, the influence of socio-economic development and health reform policies can not be ignored. Additionally, it should be noticed that CPGs are not rules, and clinicians may use all or part of particular CPGs as tools due to concrete circumstances related to care for actual patients while balancing a host of clinical considerations, as well as non-clinical considerations. Since improving the compliance of CPGs on antibacterials to help alleviate the phenomenon of AMR has been confirmed [11, 12], to further tailor effective and efficient intervention strategies, it is essential to identify key determinants among sets of factors as mentioned above, and investigate the influencing mechanism of regarding CPGs use.

According to some classical theory or framework, some personal factors and organizational factors were deemed to be the main factors to improve certain CPGs compliance [22, 23]. For example, Theory of Reasoned Action (TRA), which was the origin of many other behavioral theories (theory of planned behavior (TPB), the Health Belief Model (HBM), etc.) [24, 25], proposed that attitude and subjective norm affect behavioral intention, which directly determines people's final decision to adopt a certain behavior or not [26–29]. Moreover, organizational readiness for change (ORC) was often considered as a crucial precursor of successful implementation of complicated changes [30–32]. When organizational readiness was optimal, members of the institution would exhibit more change-related behaviors supporting the change effort. This was also in line with the job requirements or role expectations [33, 34]. However, previous studies mostly focused on the potential determinants of CPGs or health technology use behaviors from the single perspective of individuals or organizations, while there was a dearth of literature on comprehensively investigating these factors at different levels [35–39]. And some implementation theoretical frameworks or models, such as Comprehensive Framework for Implementation Research (CFIR) including multiple potential influencing factors at the level of individual, organization and so on, has confirmed the necessity and rationality for targeting multilevel determinants from an integrative insight [40–42]. Besides, traditional regression models that are mainly used in previous studies did not have advantages in simultaneously analyzing the influencing factors and the interactions between them [39, 43].

Therefore, this study aimed to construct a theoretical framework by integrating TRA and ORC theories to comprehensively investigate physicians’ CPGs use behavior in secondary and tertiary hospitals of China under the context of widely implementation of AMS program. Additionally, the regarding influencing mechanism, which consists of some potential determinants of CPGs use behavior at both the individual and organizational levels, as well as the interaction among these determinants, will be further determined by structural equation modeling (SEM).These findings not only benefit bridging the knowledge gap of overall influencing mechanism on improving the compliance of CPGs on antimicrobials, especially clarifying the acting path from the factors at the organizational-level to the ones at the individual-level, but also provide references for tailoring future strategies to expand the implementation of CPGs on antimicrobials.

## Methods

### Study design

In the context of national policy and widely implementation of AMS program, hospitals mainly audit and monitor the phenomenon that physicians prescribe on antimicrobials irrationally in key departments by establishing clinical pharmacist teams, AMS working groups, providing CPGs information and forming an information monitoring network system and other AMS management measures.

Since the irrational use of antimicrobials were rather prominent in secondary and tertiary hospitals for their mass consumption of antimicrobials, an observational, cross-sectional study was conducted in 4 secondary hospitals and 12 tertiary hospitals of eastern, central, and western China from April 2018 to Jan 2019. In this study, a secondary hospital was defined as a regional hospital with 100–499 beds, at least 0.88 health technicians and 0.4 nurses per bed, and each department has a professional title of attending physician or above[44], while a tertiary hospital was defined as a hospital with more than 500 beds, at least 1.03 health technicians and 0.4 nurses per bed, and each department has a professional title of associate chief physician or above[44].

### Theoretical model and hypotheses

**Fig. 1:**
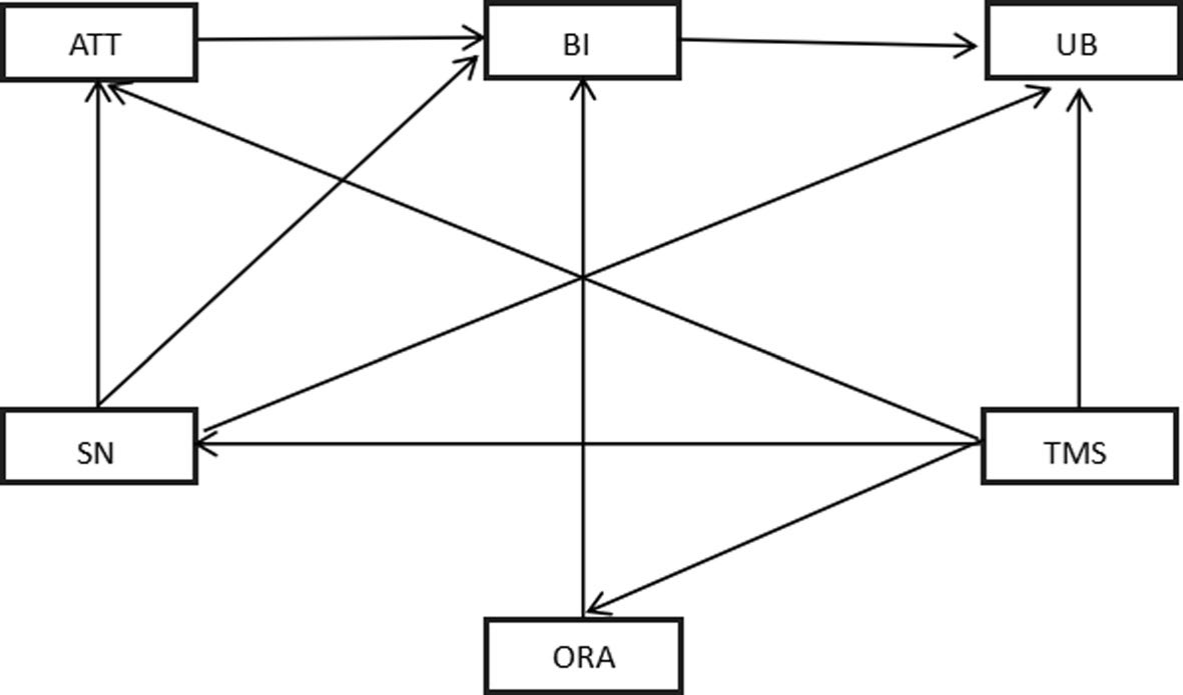
The comprehensive theoretical framework integrated by theory of reasoned action and organizational readiness for change. ATT: Attitude; SN: Subjective norms; BI: Behavioral intention; TMS: Top management support; ORA: Organizational resource allocation; UB: Use behavior

Personal behavior is usually determined by individual choice and intention, and the behavior is often constrained by management intervention in the organizational environment. Thus, this study constructed a theoretical model based on the integration of TRA and ORC. The model included five potential influencing elements of use behaviors of CPGs on antimicrobials, namely behavior attitude, subjective norms, behavior intentions, top management support, and organizational resource allocation. Figure 1 showed the proposed theoretical framework.

### Individual-level factors

In most studies in the medical field, personal use behavior is influenced by behavioral intention [25, 45], and TRA theoretical models also highlight this relationship [26]. Moreover, other studies have shown strong correlations between attitudes and intentions [46, 47]. Through social influence, key figures tend to influence other people’s behavior, and it is reported subjective norms strongly correlated with personal attitudes and behaviors. [48, 49]. While subjective norms are kinds of the perceived criteria of individual behavior and social stress [50], this may influence physicians' intentions. Based on this, the following hypotheses are proposed:

#### H1

Physicians' attitudes have a positive impact on their intentions of using CPGs on antimicrobials.

#### H2

Physicians' intentions have a positive impact on physicians' use behaviors of CPGs on antimicrobials.

#### H3

Physicians' subjective norms have a positive impact on physicians' use behaviors of CPGs on antimicrobials.

#### H4

Physicians' subjective norms have a positive impact on physicians' intention of using CPGs on antimicrobials.

#### H5

Physicians' subjective norms have a positive impact on their attitudes of using CPGs on antimicrobials.

### Organizational-level factors

Weiner BJ pointed out that organizational structures and resources shape readiness for behavior change, and it provided a more acceptable environment for organized employees [31, 34]. And organizational resources are dominated by top management support [51, 52], in the context of this study, which may influence attitudes and intentions of physicians’ use behavior. Similarly, Qureshi QA pointed out that top management support had a profound impact on subjective norms and individual use behaviors [53]. Based on this, the following hypotheses are proposed:

#### H6

Organizational resource allocation has a positive impact on physicians' intentions of using CPGs on antimicrobials.

#### H7

Top management support has a positive impact on organizational resource allocation of using CPGs on antimicrobials.

#### H8

Top management support has a positive impact on physicians' use behaviors of CPGs on antimicrobials.

#### H9

Top management support has a positive impact on physicians' attitudes of using CPGs on antimicrobials.

#### H10

Top management support has a positive impact on physicians' subjective norms of using CPGs on antimicrobials.

### Measurements

According to the theoretical framework mentioned above, as well as scales regarding TRA or ORC used in previous studies, the questionnaire of this study was developed including 18 items in six dimensions (Additional file 1). Each item was evaluated on a five-point Likert scale, where 1 = Strongly disagree, 2 = Disagree, 3 = Neutral, 4 = Agree, and 5 = Strongly agree. Additionally, the demographic characteristics of the participants, such as age, gender, professional degree, education level, were also collected.

### Sampling

As one of the largest countries in the world, China has uneven socio-economic development in various regions. Thus, a multi-stage sampling method was applied to select a certain number of hospitals from the eastern, central, and western regions of China. (1) Fujian was randomly selected as the eastern region, Hubei as the central region, and Sichuan and Yunnan as the representative provinces of the western region, respectively. (2) A total of 5*–*6 hospitals (secondary or tertiary) were randomly sampled from each region. (3) In each sampled hospital, 16*–*20 physicians of tertiary hospital and 10*–*15 physicians of secondary hospital were randomly sampled from major departments of internal medicine and surgery, respectively. And, in each tertiary hospital, 3*–*5 physicians were randomly sampled from four sorts of departments as gynecology and obstetrics, ophthalmology and otorhinolaryngology, orthopedics, and others, respectively, while a total of 10 physicians were randomly selected from all the four sorts of departments mentioned above in each secondary hospital. Thus, about 50*–*60 physicians from each sampled tertiary hospital and 30*–*40 physicians from each sampled secondary hospital were included in this study.

### Data collection

All the questionnaires were distributed by trained investigators, who explained the purpose of this study and the use of data in detail to ensure that participants understood what they needed to do and how to do it. To protect the privacy of the respondents, all responses were anonymous. Questionnaires were filled out by the participants at their convenience and returned directly to study investigators. Written informed consent was obtained from all participants.

### Data analysis

SPSS 21.0 and AMOS 21.0 software were used in this study to conduct data analysis according to the following steps. Firstly, descriptive statistical analysis was performed to describe the participants’ demographic characteristics and their measurement score of CPGs use and regarding potential predictors. Secondly, to test whether the questionnaire was acceptable, Cronbach’s α coefficient of each dimension and overall questionnaire were calculated to assess the reliability, and confirmatory factor analysis was conducted to verify the convergence and discriminant validity of the questionnaire to evaluate the consistency of the internal structure of the questionnaire. Finally, SEM was used to estimate the relationships among latent variables by path analysis and mediation effect test [54]. While path coefficients calculated by path analysis represented the direct effect, it also judged whether the hypothesis was meaningful. And the mediating effect test was applied to determine the value of indirect effects through the bootstrap method. If the value of indirect effects doesn’t contain zero in its 95% confidence interval, the indirect effect can be considered as significant [55].

## Results

### Characteristics of participants and included hospitals

A total of 822 questionnaires were dispensed and 815 valid questionnaires were returned, with a valid response rate of 99.1%. Among these participants**,** the number of males and females was 495 (56.32%) and 356 (43.68%), respectively. And 53.01% of the participants were below 35 years old. In terms of the education level, 92.39% (n = 802) of the participants had a bachelor’s degree or above. The proportions of the participants with the professional titles of junior, intermediate, and senior were 37.30%, 38.04% and 24.66%, respectively. Approximately 90% of the participants had less than 20 years in practice. Regarding the included hospitals, the sample consisted of 12 tertiary hospitals and 4 secondary hospitals. And 6 hospitals were in the eastern, 5 in the central and 5 in the western of China. (Table 1).

**Table 1:** Demographic characteristics of the participants and distribution of hospitals' ranking and regions

Variable	Category	Frequency	Percentage(%)
*Participants*			
Gender	Male	495	56.32
	Female	356	43.68
Age	< 35 years old	432	53.01
	35–45 years old	296	36.32
	> 45 years old	87	10.67
Education	Junior college or below	13	1.60
	Bachelor	345	36.32
	Master	379	46.50
	Doctor	78	9.57
Professional title	Junior	304	37.30
	Intermediate	310	38.04
	Senior	201	24.66
Years in practice	< 5 years	254	31.20
	5 ~ 10 years	241	29.60
	11-20 years	277	27.90
	21-30 years	83	10.20
	> 30 years	10	1.20
*Hospitals*			
Hospital ranking	Tertiary	12	75.00
	Secondary	4	25.00
Region	Eastern	6	37.50
	Central	5	31.25
	Western	5	31.25

### Reliability and validity

According to the results in Table 2, the Cronbach's α of the whole questionnaire was 0.930, which indicated the excellent internal consistency of the questionnaire. Among them, values of Cronbach's α in items ranged between 0.787 and 0.890, all of which were greater than the recommended threshold of 0.7 [33]. In addition, the corrected item-total correlation (CITC) value in each item was higher than the recommended threshold of 0.5 [56].

**Table 2:** Reliability test for each item, construct and the whole questionnaire

Construct	Item	CITC	Cronbach’s α
Top management support	TMS1	0.692	0.846
	TMS2	0.619	
	TMS3	0.671	
Organizational resource allocation	ORA1	0.665	0.787
	ORA2	0.547	
	ORA3	0.595	
Subjective norms	SN1	0.617	0.890
	SN2	0.643	
	SN3	0.654	
Attitude	ATT1	0.565	0.860
	ATT2	0.595	
	ATT3	0.618	
Behavioral intention	BI1	0.655	0.857
	BI2	0.670	
	BI3	0.679	
Use behavior	UB1	0.701	0.862
	UB2	0.678	
	UB3	0.662	
The whole questionnaire			0.931

Regarding the validity, Kaiser–Meyer–Olkin (KMO) and Bartlett’s test of sphericity were performed beforehand [57]. KMO values was 0.930 and the result of Bartlett's test of sphericity was significant (*P* < 0.001), indicating the appropriateness of this instrument for validity estimates. Factor loading of each item, CR and AVE of each construct were calculated to assess the convergent validity. The results showed the value of these three indicators was above the recommended value of 0.6, 0.6 and 0.7, respectively [58–60], which meant the convergent validity was acceptable (Table 3). Additionally, following the suggestion of Fornel and Larcker (1981) [61], the discriminant validity was further tested by calculating the square root of AVE. As shown in Table 4, the square root of the AVE for each construct was greater than its correlation coefficient with other constructs, except that the square root of the AVE for top management support was less than its correlation coefficient with organizational resource allocation, indicating that the discriminant validity was acceptable. (Table 4).

**Table 3:** Convergence validity test

Construct	Item	Factor loading	CR	AVE
Top management support	TMS1	0.794	0.849	0.653
	TMS2	0.777		
	TMS3	0.851		
Organizational resource allocation	ORA1	0.868	0.560	0.789
	ORA2	0.745		
	ORA3	0.610		
Subjective norms	SN1	0.807	0.735	0.892
	SN2	0.886		
	SN3	0.876		
Attitude	ATT1	0.758	0.862	0.676
	ATT2	0.844		
	ATT3	0.861		
Behavioral Intention	BI1	0.798	0.859	0.671
	BI2	0.847		
	BI3	0.811		
Use behavior	UB1	0.784	0.679	0.864
	UB2	0.835		
	UB3	0.852

**Table 4:** Discriminant validity test

Construct	TMS	SN	ATT	ORA	BI	UB
TMS	**0.808**					
SN	0.510	**0.945**				
ATT	0.476	0.701	**0.822**			
ORA	0.886	0.452	0.422	**0.888**		
BI	0.573	0.643	0.678	0.573	**0.819**	
UB	0.688	0.589	0.555	0.637	0.722	**0.929**

The square root of the AVE for each construct was in bold

### Measurement scores of use behaviors and the predictors

Physicians' use behaviors included a range of initiatives to CPGs on antimicrobials over the past year. The mean score of participants’ use behaviors of CPGs on antimicrobials was 3.95, with a majority (77.67%) of the behavior score above neutral. Specifically, the score of whether participants strictly abide by CPGs in clinical work was 3.98, learning about actively participating in CPGs on antibacterials was 3.99, and the use of guidelines to promote peripheral colleagues also scored up to 3.87.

For the predictors at the organizational-level, top management support and organizational resource allocation had a mean score of 3.99 and 3.87, respectively. Regarding predictors at the individual-level, the scores of subjective norms (Mean = 4.15, SD = 0.67), attitudes (Mean = 4.29, SD = 0.63), and behavior intentions (Mean = 4.13, SD = 0.64) were relatively high, which revealed a strong tendency in favor of the use of CPGs on antimicrobials. The proportion of participants who scored above the neutral was ranged from 74.36% to 92.02% among all the variables. (Table 5).

**Table 5:** Measurement scores of the participants

Dimension	Mean	SD	Median	N (%) of scores > 3
Top management support	3.99	0.71	4	659 (80.86)
Organizational resource allocation	3.87	0.80	4	606 (74.36)
Subjective norms	4.15	0.67	4	718 (88.10)
Attitude	4.29	0.63	4	750 (92.02)
Behavioral intention	4.13	0.64	4	723 (88.71)
Use behaviour	3.95	0.70	4	633 (77.67)

### Structural equation modeling

The overall model fit index of the hypothetical model was as follows: χ^2^/df = 3.130 (< 5), GFI = 0.949 (> 0.9), AGFI = 0.931 (> 0.9), RMSEA = 0.051 (< 0.08), and CFI = 0.971 (> 0.9), which suggested that the model fit the data well.

**Fig. 2:**
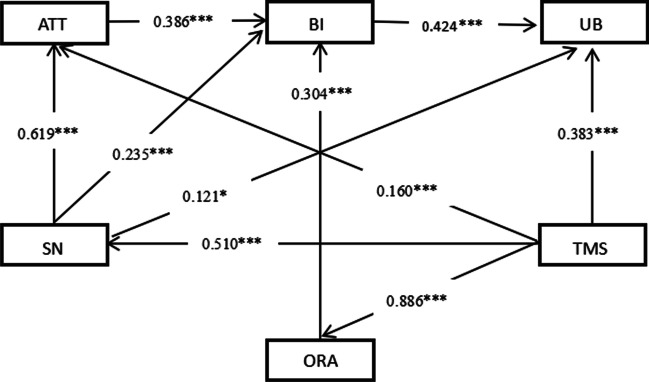
Determinants of physicians’ use behaviors of CPGs on antimicrobials. *p < 0.05; **p < 0.01; ***p < 0.001. ATT: Attitude; SN: Subjective norms; BI: Behavioral intention; TMS: Top management support; ORA: Organizational resource allocation; UB: Use behavior.

The final hypothesized model with the standardized estimates among the constructs was shown in Fig. 2 and Table 6. Totally, 64.75% of the variance was explained and all the hypotheses were verified by the model. Regarding the direct determinants of physicians’ use behaviors of CPGs on antimicrobials, the model indicated that top management support in favor of CPG on antimicrobials was linked to regarding CPG use at organizational-level (*β* = 0.383, *P* < 0.001). And at the individual-level, behavioral intentions in favor of use CPGs were linked to higher physicians’ use behaviors of CPGs on antimicrobials (*β* = 0.434, *P* < 0.001), and subjective norms were linked to lower physicians' use of CPGs on antimicrobials (*β* = 0.112, *P* < 0.001)..

**Table 6:** Results of structural equation modeling analysis

Paths	Direct effect	Indirect effect	The total effect
Top management support → Subjective norms	0.510***	0	0.510***
Top management support → Attitude	0.160***	0.316***	0.476***
Top management support → Organizational resource allocation	0.886***	0	0.886***
Top management support → Behavioral Intention	0	0.573***	0.573***
Top management support → Use behavior	0.383***	0.305***	0.688***
Organizational resource allocation → Behavioral Intention	0.304***	0	0.304***
Organizational resource allocation → Use behavior	0	0.129***	0.129***
Attitude → Behavioral Intention	0.386***	0	0.386***
Attitude → Use behavior	0	0.164***	0.164***
Subjective norms → Attitude	0.619***	0	0.619***
Subjective norms → Behavioral Intention	0.235***	0.239***	0.474***
Subjective norms → Use behavior	0.121*	0.201***	0.322***
Behavioral Intention → Use behavior	0.424***	0	0.424***

*p < 0.05; **p < 0.01; ***p < 0.001

Overall, on the behavior of using CPG on antimicrobials, top management support had the strongest total effect (0.688), followed by behavioral intentions (0.424) and subjective norms (0.322). In addition, there were some variables had a mediating effect on use behavior of CPGs on antimicrobial. The indirect effect from high to low was top management support (0.305), subjective norms (0.201), attitudes (0.164), and organizational resource allocation (0.129), respectively.

## Discussion

To bridge the knowledge gap of the overall influencing mechanism on the physicians’ use of CPGs on antimicrobial, this study investigated the potential influencing factors at the individual-level and organizational-level by integrating TRA and ORC. Additionally, it applied to SEM approach to simultaneously identify the direct and indirect effects of the five potential influencing factors on the final CPGs use behaviors, as well as the interaction between these factors. The findings of this study revealed that behavioral attitudes, subjective norms, behavioral intentions, top management support and organizational resource allocation all had significant impacts on the use of CPGs on antimicrobials. Behavioral intentions at the individual-level and the support of the managers at the organizational-level were found to have a substantial direct effect. All the hypotheses were tested in the extended model, indicating that the integration of ORC and TRA well explained the underlying mechanism for the use of CPGs on antimicrobials in the studied population.

At individual-level, consistent with previous research [62–64], this study verified that behavioral intention was one of the most important predictors leading to use behavior. Subjective norms and behavioral attitudes directly influenced physicians' intention to use CPGs on antimicrobials, and indirectly influenced the final use behavior. Since the attitude and subjective norms are often related to a sense of security [63], which are to some extent shaped by the external pressure physicians feel, the important roles of attitudes and subjective norms playing on CPGs use behaviors are plausible. This finding indicated that it was important to promote the physicians' positive attitudes to the use of CPGs on antimicrobials. What is more, due to the significant direct impacts of subjective norm on both attitude and behavior of using CPGs on antimicrobials, it also highlighted the importance of recalling the demonstration role of key figures during the process of expanding certain CPGs implementation, whose perceptions or behavior toward certain CPGs or other health technologies would form a kind of perceived norm and pressure on the other physicians around them [64].

For the potential influencing factors at the organizational-level, this study demonstrated the important role of top management support in the overall influencing mechanism on application behavior of CPGs on antimicrobials. On the one hand, the significant impact of top management support on organizational resource allocation was reported in this study, which verified the hypothesis proposed according to the ORC theory at the organizational level. Since top management support often includes special funding, training and guidance provided by senior managers of health institutions, it is not hard to understand that these supports will be very important for the organization to implement new strategies [65, 66], and inevitably lead to a realignment of the allocation of resources within the organization. On the other hand, significant effects were also detected among top management support and various factors at the individual-level, namely physicians’ attitudes, subjective norms and use behaviors on the CPGs of antimicrobials. As reported by Qureshi QA et al. [53], hospital managers played an important role in formulating the vision of organizational development, developing organizational culture and shaping the physicians’ expected behavior and norms. In terms of promoting the use of CPGs on antimicrobials, hospital managers are capable of exerting direct or indirect impact on physicians' attitudes, perceptions and intentions towards CPGs use by establishing a series of supportive incentives and monitoring system, which will subsequently drive physicians to adjust their antimicrobials prescription behaviors according to the norms recognized by the organization.

Besides, the indirect effect of organizational resource allocation on the CPGs use behavior through intention was also demonstrated in this study, which was a noteworthy point easily be ignored by previous studies merely focusing on factors at the organizational level [18, 67]. The plausible reason may be that the physicians tend to assess the resource they possess to implement the CPGs before they finally take certain actions. The results reminded us that abundant organizational resource allocation is one of the prerequisites for the effective implementation of CPGs on antimicrobials, which can be also deemed as the "catalyst" to stimulate the activity of the physicians.

## Implication for practice

Based on the identified overall influencing mechanism of the physicians’ use behaviors of CPGs on antimicrobials, and combined with currently universal interventions for antimicrobial stewardship, some measures are recommended for further promoting the implementation of certain CPGs.

On the one hand, some explicit supporting approaches should be applied or strengthened within the hospitals. Firstly, hospital managers are advised to allocate special organizational resource for expanding the use of CPGs on antimicrobials, which includes but not limited to special regulation, funding, personnel or departments. For example, developing and regularly updating guidelines on antimicrobial therapy, formulating sets of antimicrobial prescription at hospital level, establishing a guideline group for drug use at the department level to guide the appropriate use of antibiotics in specific clinical scenario, and so on. Secondly, seminars, lectures or special training, and other channels of information dissemination are essential for advancing the knowledge of certain CPGs. In view of the important role-played by pharmacists in AMS [68], regular seminars and lectures were recommended to hold to strengthen cooperation and communication between pharmacists and physicians. Especially for key departments with large consumption amount of antibiotics, carrying out specialized training on CPGs of antimicrobials use is also in urgent need. Thirdly, to overcome the barriers of CPGs use in a timely manner, it is essential to establish a monitoring and feedback mechanism. For instance, hospital managers can regularly collect and analyze antibiotic prescription data, and invite experts and front-line medical staff to investigate the problems underlying the current status of implementation and compliance of the CPGs on antimicrobials. Some adjustment can be made to regarding CPGs to improve their feasibility and operability in clinical practice. Also, by regularly evaluating the CPG compliance of physicians, incentive measures can be adopted to encourage medical staff to continue to comply with CPGs on antimicrobials and promote rational use of antibiotics. By agency of these concrete means with substantial organizational resource allocated, a comprehensive long-term mechanism will be developed, which will benefit the physicians fully realizing the firm will of the hospital in promoting the use of CPGs on antimicrobials, as well as promote their consciously adhering to certain CPGs during the prescription.

On the other hand, since the important role of subjective norm was confirmed in many studies, implicit approaches to promote the behavior-driven effect of subjective norms are also recommended, which attach importance to the demonstration effect of the influential people for physicians (generally referred to experts or professors in their field, hospital managers and senior colleagues, etc.). Specifically, these influential people can be mobilized to deliver experience-sharing sessions on their use of CPGs on antimicrobials, and the form of mentor-apprentice teaching can be awarded. Thus, the enthusiasm of physicians to follow the CPGs compliance behavior can be mobilized through the active advocacy of these influential people. In addition to the exemplary role of prominent figures, the influence of group members' behavioral characteristics on individual behaviors has also attracted widespread attention in recent years, and specific social norm feedback interventions have also been formulated. For example, by informing certain physician that his or her antimicrobials consumption amount has reached the top 20% of doctors in similar clinical departments, expose their behavior status to the group comparison, which can trigger individual changes in knowledge, attitude and belief, and finally lead to changes of their antibiotics prescription. All these measures will help physicians develop social norms and beliefs about CPGs implementation, which also benefits maintaining and reinforcing their adherence to CPGs on antimicrobials among the physician community.

Furthermore, it must be noted that impact of macro-policy at the national or local level can not be ignored. Under the context of the in-depth reform of the medical and health system in China, the implementation and compliance of CPGs on antimicrobials can be further promoted steadily during the process of many special actions, such as eliminating adverse economic incentives, standardizing the prescription behavior of medical staff, strengthening medical quality management and so on [17, 18, 69]. However, as reported by some scholars, some management strategies were not effective in reducing the irrational use of antibiotics[14, 15], which may imply that it is unwise to rely solely on macro policies and do nothing about the management of CPGs at the medical institution level.

### Strengths and limitations

In addition to implication for practice, this study was also strengthened by some features as follows. Firstly, the research model of this study was derived from the integration of TRA and ORC theories, which has demonstrated good explanatory power and provided comprehensive insight to investigate the overall influencing mechanism of corresponding CPGs use. Secondly, the application of SEM enabled this study to determine the influencing factors of final behavior and to investigate the interaction between these factors at the individual-level and organizational-level simultaneously, which made the research results more robust. Finally, the sample areas included provinces from the eastern, central and western parts of China, respectively, which would benefit the representativeness of the study.

However, there were still some limitations. Firstly, since all data were obtained by self-reported, the bias of social desirability can not be eliminated that the participants tend to give more positive assessments on themselves and the working institutions. Secondly, due to time and fund constraints, only a limited number of potential influencing factors were included in this study, which may fail to cover all the clinical and non-clinical factors affecting use behavior of CPGs on antimicrobials. Additionally, TRA applied has been advanced since its development in the 1970s by including more variables to fill the intention-behavior gap, thus, ignoring the newly added variables may impair the explanation power of the integrated model in this study. At the same time, this study investigated the influencing mechanism simply from the individual and organizational levels, but we can’t rule out the possibility that other influencing mechanism exist. Finally, cross-sectional studies have their limitations in causal inference, and the cluster effect may exist due to the difference in physicians’ CPGs compliance between hospitals with different rankings and location region.

### The future research direction

Firstly, the measurement of CPGs compliance behavior could be more objective in future studies, and it is also recommended to obtain real-world data on antimicrobials prescription from public databases or hospital information system. Secondly, more variables, especially those newly added from the advanced theory, should be included in future studies, such as perceived behavioral control, social support, self-efficacy and so on. At the same time, it is also strongly recommended to include some other potential determinants, such as policy environment, patient factors, applicability of guidelines, to further investigate other potential influencing mechanism on CPGs use behavior. Furthermore, since there is no context-free way to assess inappropriate antimicrobials use, future studies are recommended to select a typical clinical scenario to assess physicians’ compliance to CPGs on antimicrobials, such as the treatment of upper respiratory tract infection, and so on. Finally, regarding to the methodology, future research would conduct multi-level analysis to deal with cluster effect, and it can also extract samples at different time points to form panel data, so as to infer the influencing factors more rigorously.

## Conclusions

By integrating TRA and ORC theories, this study investigated the influencing mechanism for the use of CPGs on antimicrobials from the individual and organizational perspectives. SEM approached was applied to verify the proposed theoretical framework. The findings of this study showed the significance of multiple factors relevant to the use of CPGs on antimicrobials, including behavioral attitudes, subjective norms and behavior intentions at the individual-level, as well as top management support and organizational resource allocation at the organizational-level. These findings will not only advance the knowledge of the overall influencing mechanism on the application behavior of CPGs on antimicrobials, clarify the interactions among factors at the individual-level and organizational-level, but also benefit tailoring future strategies for promoting the use of CPGs on antimicrobials, and providing clues to more efficient prescription interventions.

## Supplementary Information

**Additional file 1**. Questionnaire. The questionnaire represents the data collection instrument that was developed for this study, hasn’t previously been published elsewhere.

## Data Availability

The datasets generated during and/or analyzed during the current study are available from the corresponding author on reasonable request.

## Abbreviations

AMR: Antimicrobial resistance
AMS: Antimicrobial stewardship
CPGs: Clinical practice guidelines
TRA: Theory of reasoned action
ORC: Theory of organizational readiness for change
SEM: Structural equation modeling
ORA: Organizational resource allocation
UB: Use behavior
