# Supplementary material for: Influencing mechanism of the use behavior of clinical practice guidelines on antimicrobials: evidence from the integration of theory of reasoned action and organizational readiness for change
Source: BMC Med Inform Decis Mak. 2022 Oct 26;22:279. doi: 10.1186/s12911-022-02019-w (PMC9598025; doi:10.1186/s12911-022-02019-w)
Supplement: Supplementary file 1 — Additional file 1. Questionnaire. The questionnaire represents the data collection instrument that was developed for this study, hasn’t previously been published elsewhere. [file 12911_2022_2019_MOESM1_ESM.doc]

**Research questionnaire for determinants of physicians’**

**utilization behaviors of CPG on antimicrobials**

**Part 1. Structural Scales**

Notes. There are 5 numbers (1, 2, 3, 4, 5) on the right side of each item, where “1” means “Strongly disagree”, “2” means “Disagree”, “3” means “Neutral”, “4” means “Agree”, and “5” means “Strongly agree”. Please tick or circle the number that best fits your real feelings on the item.

**Domain: Organizational Level**

| ***Top management support*** | | | | | |
| --- | --- | --- | --- | --- | --- |
| Administrators promote the widely use of CPG on antimicrobials in various departments. | 1 | 2 | 3 | 4 | 5 |
| Administrators provide supports in training, funding, etc. | 1 | 2 | 3 | 4 | 5 |
| Administrators attach great importance to the promotion of CPG on antimicrobials. | 1 | 2 | 3 | 4 | 5 |
| ***Organizational Resource Allocation*** | | | | | |
| The hospital provides information about CPG on antimicrobials. | 1 | 2 | 3 | 4 | 5 |
| The hospital performs daily inspection, supervision and evaluation. | 1 | 2 | 3 | 4 | 5 |
| The hospital holds regular feedback on the use of CPG on antimicrobials. | 1 | 2 | 3 | 4 | 5 |

**Domain: Individual Level**

| ***Subjective Norm*** | | | | | |
| --- | --- | --- | --- | --- | --- |
| People who are important to me tend to follow CPG on antimicrobials. | 1 | 2 | 3 | 4 | 5 |
| People who are important to me have a positive evaluation of CPG on antimicrobials. | 1 | 2 | 3 | 4 | 5 |
| People who are important to me think it’s a right thing to use CPG on antimicrobials. | 1 | 2 | 3 | 4 | 5 |
| ***Attitude*** | | | | | |
| I think it’s a right thing to follow the CPG on antimicrobials. | 1 | 2 | 3 | 4 | 5 |
| I think it’s a wise choice to follow the CPG on antimicrobials. | 1 | 2 | 3 | 4 | 5 |
| I think it’s good for all to follow the CPG on antimicrobials. | 1 | 2 | 3 | 4 | 5 |
| ***Behavioral Intention*** | | | | | |
| I am willing to use CPG on antimicrobials. | 1 | 2 | 3 | 4 | 5 |
| I will follow the CPG on antimicrobials in the future. | 1 | 2 | 3 | 4 | 5 |
| I am willing to recommend CPG on antimicrobials to other doctors. | 1 | 2 | 3 | 4 | 5 |
| ***Utilization behavior*** | | | | | |
| In the past year, I have strictly followed the CPG on antimicrobials in practice. | 1 | 2 | 3 | 4 | 5 |
| In the past year, I have actively participated in the study or training of CPG on antimicrobials. | 1 | 2 | 3 | 4 | 5 |
| In the past year, I have actively recommended the CPG on antimicrobials to colleagues. | 1 | 2 | 3 | 4 | 5 |

**Part 2. Basic information survey card**

1. Please choose your gender. ________ A. Male B. Female

2. Please write down your age: ________

3. Please choose your educational level. ________

A. Junior college or below B. Bachelor C. Master D. Doctor

4. Please choose your professional title. ________

A. Junior B. Intermediate C. Senior

5. Please write down your department: ________

6. Please choose your years in practice.

A. <5 years B. 5~10 years C. 11~15years D. 16~20 years E. >20 years
